# Total burden of cerebral small vessel disease predict subjective cognitive decline in patients with Parkinson’s disease

**DOI:** 10.3389/fnagi.2024.1476701

**Published:** 2024-11-22

**Authors:** Wenchao Qiu, Weili Hu, Yingchao Ge, Peiting Liu, Minghui Zhao, Haifeng Lu, Jian Tao, Shouru Xue

**Affiliations:** ^1^Department of Neurology, The First Affiliated Hospital of Soochow University, Suzhou, China; ^2^Department of Neurology, The Affiliated Huai’an Hospital of Xuzhou Medical University, Huai’an, China; ^3^Department of Neurology, Lianshui County People’s Hospital, Huai’an, China; ^4^Department of Neurology, Qidong People’s Hospital, Nantong, China; ^5^Department of Radiology, The Affiliated Huai’an Hospital of Xuzhou Medical University, Huai’an, China

**Keywords:** Parkinson’s disease, subjective cognitive decline, cerebral small vessel disease, magnetic resonance imaging, predictive biomarkers

## Abstract

**Introduction:**

This study investigates the correlation between the total burden of Cerebral Small Vessel Disease (CSVD) and Subjective Cognitive Decline (SCD) in patients with Parkinson’s disease (PD).

**Methods:**

A cross-sectional design was employed, involving 422 patients with PD. Demographic and clinical data were collected. Brain magnetic resonance imaging (MRI) was conducted to identify CSVD markers. SCD was assessed using the Cognitive Complaints Inventory (CCI).

**Results:**

Logistic regression analyses revealed that the total burden of CSVD and specific imaging markers, including Deep White Matter Hyperintensities (DWMH), Periventricular Hyperintensities (PVH), and Enlarged Perivascular Spaces (EPVS), were significant predictors of SCD. The total burden of CSVD demonstrated the highest predictive accuracy for SCD in PD patients.

**Discussion:**

The findings suggest that the total burden of CSVD, as measured by MRI, could serve as a potential biomarker for early identification of cognitive decline in PD, highlighting the importance of considering vascular factors in the early detection of cognitive changes in PD.

## 1 Introduction

Parkinson’s disease (PD) is a progressive neurodegenerative disorder, initially characterized by motor symptoms such as tremor, rigidity, and bradykinesia. However, the non-motor symptoms, particularly cognitive decline, have gained attention for their significant impact on patient’s quality of life (Deliz et al., 2024). The cognitive manifestations in PD are multifaceted and can present as both objective deficits and subjective experiences, with the latter often preceding the clinical diagnosis of dementia (Hely et al., 2008).

Subjective Cognitive Decline (SCD) in PD refers to the self-reported worsening of cognitive faculties, such as memory and attention, which may not necessarily align with objective cognitive testing (Jessen et al., 2014; Cacciamani et al., 2021). Increasing evidence suggests that in the elderly, SCD may signal the onset of a non-standard cognitive deterioration, even in the absence of measurable cognitive impairment or depressive symptoms, potentially leading to the development of dementia (Rabin et al., 2015). Identifying SCD is crucial as it potentially signals the early stages of cognitive impairment and provides an opportunity for intervention. Early detection of SCD in PD is vital for implementing timely therapeutic strategies that may ameliorate disease progression (Huang et al., 2023; Hong and Lee, 2023; Flores-Torres et al., 2023).

Cerebral Small Vessel Disease (CSVD) has been implicated in the cognitive trajectory of neurodegenerative diseases (Kim et al., 2022; Wardlaw et al., 2013). It may contribute to cognitive decline by affecting information processing speed and executive function (Prins et al., 2005). This involves a spectrum of pathological processes that affect the brain’s microvasculature, leading to white matter hyperintensities, lacunes, and other radiological features (Wardlaw et al., 2013).

Recently, a comprehensive score that encompasses all the aforementioned markers has been implemented to evaluate the overall impact of CSVD, further examining the influence of total burden of CSVD on cognitive function (Li et al., 2021; Fan et al., 2021; Staals et al., 2015; del Brutto et al., 2018). There have also been reports on the role of CSVD in cognitive impairment associated with PD (Ma et al., 2021; Chen et al., 2023). However, there is a lack of research focusing on the association between the total burden of CSVD and SCD in patients with PD. This study aims to explore the association between the total burden of CSVD and SCD in patients with PD. We hypothesize that a greater burden of CSVD will be correlated with a higher prevalence of SCD, indicating CSVD’s potential as a biomarker for early cognitive decline in PD.

## 2 Materials and methods

### 2.1 Participants

This study employed a cross-sectional design to investigate the relationship between the total burden of CSVD and subjective cognitive decline in patients with PD. The research protocol was approved by the Ethics Committees of the First Affiliated Hospital of Soochow University and the Ethics Committees of the Affiliated Huai’an Hospital of Xuzhou Medical University. All participants provided written informed consent prior to enrollment. Patients were diagnosed with Parkinson’s disease according to the Movement Disorder Society (MDS) Clinical Diagnostic Criteria (Postuma et al., 2015). Inclusion criteria included age over 50 years and participants must be capable of engaging in and completing cognitive function assessments.

**Figure 1:**
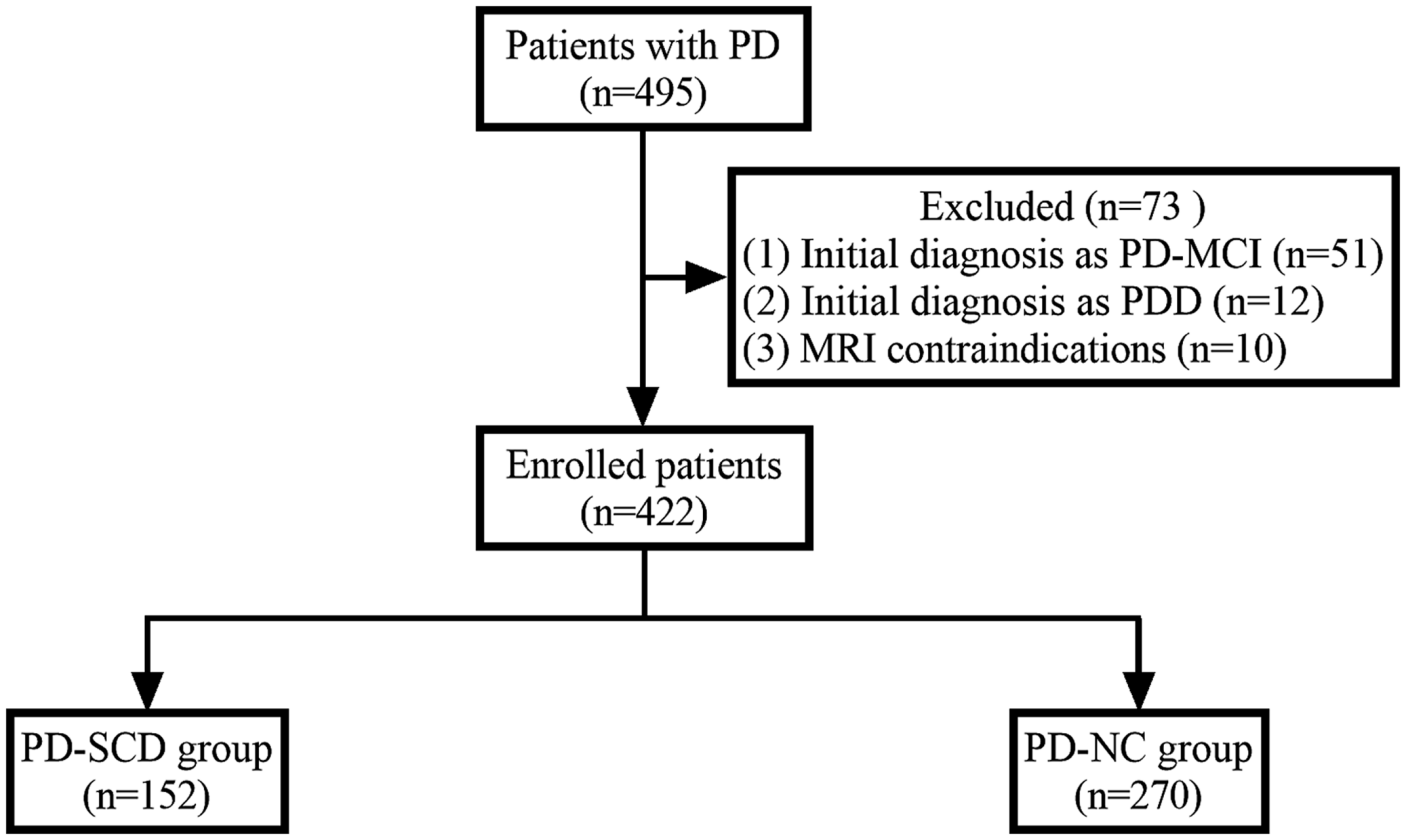
Flowchart of patient recruitment. PD, Parkinson’s disease; PD-MCI, PD with mild cognitive impairment; PDD, PD with dementia; PD-SCD, PD with subjective cognitive decline; PD-NC, PD with normal cognition.

Participants were excluded based on several criteria to ensure study integrity: (1) Presence of contraindications for MRI scanning, such as implanted pacemakers or metallic devices. (2) Patients with a history of other neurodegenerative diseases, cerebrovascular accidents, or cerebral structural abnormalities. (3) A documented history of severe psychiatric disorders that could potentially skew study outcomes. (4) PD patients with existing MCI or dementia were excluded based on established diagnostic criteria (Litvan et al., 2012). For detailedly, MCI in PD was diagnosed according to the Movement Disorder Society (MDS) Task Force criteria for PD-MCI Level I (Litvan et al., 2012). For the diagnosis of Dementia in PD (PDD), we employed the Chinese version of the Parkinson’s Disease Cognitive Rating Scale (PD-CRS). The specific cutoff score for diagnosing PDD was established at 73.5, aligning with validated norms for the Chinese population (Tan et al., 2020; Pagonabarraga et al., 2008). A total of 495 patients were initially screened. After applying the exclusion criteria, patients with MCI (*n* = 51), dementia (*n* = 12), and MRI contraindications (*n* = 10) were excluded, resulting in a final study cohort of 422 participants. Figure 1 depicts the flowchart of patient recruitment.

Demographic and clinical data were meticulously collected for all participants, ensuring a comprehensive understanding of each individual’s health profile. This included age, sex, education level, disease duration, and levodopa-equivalent dosage. Additionally, we documented the presence of comorbid conditions such as hypertension, diabetes mellitus, and hyperlipidemia, as well as lifestyle factors like smoking and drinking habits. The severity of motor symptoms was quantified using the Movement Disorder Society Unified Parkinson’s Disease Rating Scale (MDS-UPDRS III), and cognitive function was evaluated with the Montreal Cognitive Assessment (MoCA) score.

### 2.2 MRI acquisition and analysis

All participants underwent brain magnetic resonance imaging (MRI) using a Siemens 3.0 T scanner (Erlangen, Germany) equipped with a 32-channel head coil. The imaging protocol included axial T1-weighted imaging (T1WI) with the following parameters: repetition time (TR)/echo time (TE) of 2,300/2.98 ms, flip angle of 8 degrees, field of view (FOV) of 256 × 256 mm, matrix size of 256 × 256, and a slice thickness of 1 mm. Axial T2-weighted imaging (T2WI) was performed with TR/TE of 5,000/98 ms, flip angle of 150 degrees, FOV of 220 × 220 mm, matrix size of 512 × 512, and a slice thickness of 3 mm. Fluid-attenuated inversion recovery (FLAIR) imaging used TR/TE of 9000/73 ms, flip angle of 150 degrees, FOV of 220 × 220 mm, matrix size of 512 × 512, and a slice thickness of 3 mm. Diffusion-weighted imaging (DWI) was acquired with TR/TE of 8,900/91.2 ms, flip angle of 192 degrees, FOV of 192 × 192 mm, matrix size of 192 × 192, and a slice thickness of 2.0 mm with b-values of 0 and 1,000 s/mm^2^. Susceptibility-weighted imaging (SWI) utilized TR/TE of 1,230/13.4 ms, flip angle of 25 degrees, FOV of 206 × 206 mm, matrix size of 256 × 256, and a slice thickness of 1.5 mm.

Two trained neuroradiologists (Jian Tao and Haifeng Lu) were employed to independently evaluated the MR images. Both of them were blinded to the clinical data. The CSVD markers, including silent lacunar infarction (SLI), cerebral microbleeds (CMBs), deep white matter hyperintensities (DWMH), periventricular hyperintensities (PVH) and enlarged perivascular spaces (EPVS) in basal ganglia (BG-EPVS) and centrum semiovale (CS-EPVS), were defined and rated according to the STRIVE criteria (Wardlaw et al., 2013). In order to evaluate the interrater reliability, the Cohen’s Kappa coefficient was calculated in our study. The interrater reliability was excellent for all neuroimaging biomarkers (κ = 0.837, SLI; κ = 0.829, CMBs; κ = 0.884, DWMH; κ = 0.896, PVH; κ = 0.897, BG-EPVS and κ = 0.871, CS-EPVS). Discrepancies in the evaluations were resolved by deferring to the judgment of the senior neuroradiologist, Haifeng Lu, whose interpretations were definitive for the study’s analytical framework. Finally, the total burden of CSVD was quantified using a validated scoring system ranging from 0 to 4, with each of the following parameters contributing one point to the total score (Staals et al., 2014): presence of one or more SLIs; presence of one or more CMBs; either DWMH with a Fazekas score of 2–3 or PVH extending into the deep white matter with a Fazekas score of 3; moderate to severe (grade 2–4) PVS in the basal ganglia.

### 2.3 Subjective cognitive decline (SCD) assessment

SCD was evaluated using the Chinese translation version of the Cognitive Complaints Inventory (CCI) (Thomas-Antérion et al., 2006). It consists of 10 questions designed to detect alterations in cognitive performance within a six-month retrospection (for details, refer to Supplementary Table S1). The CCI is rater-administered and does not require caregiver input. The subjects were asked to read the questions and answer with either yes or no. A threshold score above 3 on the CCI is considered diagnostic of SCD (Yoo et al., 2020).

### 2.4 Statistical analysis

Data were analysed using SPSS (version 26.0). Demographic and neuroimaging characteristics were compared between PD patients with SCD and without SCD. For this purpose, we utilized Chi-square tests to analyse categorical data, *t*-tests for variables that were normally distributed, and Mann–Whitney U tests for non-parametric data to ensure a robust statistical approach. We did univariate and multivariate linear analysis with CCI scores as dependent variables. For the multivariate model, variables that demonstrated significance in the univariate analysis at the *p*<0.05 level were selectively integrated. To pinpoint potential predictors for SCD, we employed logistic regression analysis. Consistent with our approach in the linear regression, only variables that achieved significance in the univariate analysis were incorporated into the multivariate logistic regression model. This strategy was adopted to streamline the model’s complexity and mitigate the risk of overfitting. The odds ratios (OR) and their 95% confidence intervals (CI) were computed, and associations were considered statistically significant for *p*-values less than 0.05. The predictive accuracy of different CSVD markers for SCD in PD patients was evaluated using the area under the curve (AUC) from the receiver operating characteristic (ROC) curves. ROC curves were generated using the GraphPad Prism (version 9.5). To compare the efficacy of different CSVD markers in predicting SCD in patients with PD, the DeLong test was employed to compare the differences in AUC values.

## 3 Results

The study included a total of 422 participants with PD, after excluding those with mild cognitive impairment or dementia, as well as those with MRI contraindications. Among this population, 152 participants reported a CCI score surpassing the threshold of 3, denoting the presence of SCD (for details, refer to Table 1).

**Table 1:** Demographic and neuroimaging characteristics of the patients with Parkinson’s disease.

	PD with SCD	PD without SCD	*p* value
	*n* = 152	*n* = 270	
Demographic and clinical characteristics			
Age, y	64.66 ± 6.46	63.11 ± 7.62	0.0357
Age at onset, y	63.08 ± 6.73	60.68 ± 6.68	<0.0001
Sex (Male)	107 (70.4%)	134 (49.6%)	<0.0001
Education, y	5.92 ± 2.75	7.95 ± 2.98	<0.0001
Disease duration, m	25.09 ± 12.39	26.14 ± 12.42	0.8267
Levodopa-equivalent dose, mg	196.09 ± 104.38	166.24 ± 95.80	0.0001
Hypertension	43 (28.3%)	57 (21.1%)	0.0959
Diabetes mellitus	6 (3.9%)	6 (2.2%)	0.3061
Hyperlipidemia	17 (11.2%)	45 (16.7%)	0.1267
Smoking	29 (19.1%)	46 (17.0%)	0.5984
Drinking	21 (13.8%)	35 (13.0%)	0.8042
MDS-UPDRS III score	18.07 ± 2.29	16.07 ± 2.05	<0.0001
MoCA score	28.03 ± 0.59	27.68 ± 0.58	<0.0001
Imaging findings			
SLI	69 (45.4%)	55 (20.4%)	<0.0001
CMBs	50 (32.9%)	39 (14.4%)	<0.0001
DWMH	0.82 ± 0.53	0.57 ± 0.52	<0.0001
PVH	1.03 ± 0.58	0.71 ± 0.45	<0.0001
CS-EPVS	2.36 ± 0.66	2.13 ± 0.78	0.0001
BG-EPVS	1.95 ± 0.48	1.58 ± 0.63	<0.0001
Total CSVD score	1.78 ± 0.81	0.90 ± 0.59	<0.0001

SCD, Subjective Cognitive Decline; y, years; m, months; MDS-UPDRS, Movement Disorder Society Unified Parkinson’s Disease Rating Scale; MoCA, Montreal Cognitive Assessment; SLI, silent lacunar infarction; CMBs, cerebral microbleeds; DWMH, deep white matter hyperintensities; PVH, periventricular hyperintensities; CS-EPVS, enlarged perivascular spaces of centrum semioval; BG-EPVS, enlarged perivascular spaces of basal ganglia; CSVD, cerebral small vessel disease.

The mean age of the PD patients with SCD was 64.66 years, which was slightly higher than those without SCD. Moreover, the age at onset, males, and levodopa-equivalent dosage were significantly different between PD with SCD and those without SCD. Notably, the PD with SCD cohort had a lower average education of 5.92 years compared to 7.95 years in the PD without SCD group. Regarding the clinical features, the presence of hypertension and diabetes mellitus did not differ significantly between the two groups. However, the PD with SCD group showed a more pronounced severity as measured by the MDS-UPDRS III score and the MoCA score. In terms of neuroimaging findings, the PD with SCD group exhibited a higher prevalence of SLI, CMBs, and more severe scores for DWMH, PVH, CS-EPVS, and BG-EPVS. The total CSVD score was also significantly higher in the PD with SCD group.

In linear analysis with CCI scores as dependent variables, age at onset, sex, education, MDS-UPDRS III scores, and MoCA scores were significantly associated with CCI scores. Notably, several neuroimaging markers of CSVD, including SLI, DWMH, PVH, CS-EPVS and total burden of CSVD, showed significant associations with CCI scores, suggesting that these markers are robust predictors of cognitive decline even after controlling for other variables (Supplementary Table S2).

The univariate logistic regression analysis revealed significant associations between the presence of SCD in our PD cohort and demographic and clinical characteristics, including age, age at onset, males, education, disease duration, levodopa-equivalent dosage, presence of hypertension, MDS-UPDRS III score and MoCA score. Furthermore, each CSVD marker demonstrated a significant association with SCD in the univariate context (for specifics, refer to Table 2).

**Table 2:** Logistic regression analysis for clinical and imaging predictors of SCD.

	Univariate analysis		Multivariate analysis	
	OR (95%CI)	*p* value	OR (95%CI)	*p* value
Demographic and clinical characteristics				
Age, y	1.030 (1.002–1.060)	**0.037**		
Age at onset, y	1.055 (1.024–1.089)	**0.0006**	1.066 (1.015–1.119)	**0.011**
Sex				
Female	Ref		Ref	
Male	2.413 (1.590–3.704)	**<0.0001**	3.184 (1.650–6.144)	**0.0006**
Education, y	0.7837 (0.7242–0.8444)	**<0.0001**	0.765 (0.682–0.858)	**<0.0001**
Disease duration, m	0.9931 (0.9772–1.009)	0.3996		
Levodopa-equivalent dose, mg	1.003 (1.001–1.005)	**0.0035**	1.004 (1.000–1.007)	**0.024**
Hypertension	1.474 (0.9295–2.329)	0.0969		
Diabetes mellitus	1.808 (0.5564–5.877)	0.3125		
Hyperlipidemia	0.6296 (0.3383–1.125)	0.1291		
Smoking	1.148 (0.6811–1.911)	0.5985		
Drinking	1.076 (0.5937–1.911)	0.8042		
MDS-UPDRS III score	1.535 (1.382–1.717)	**<0.0001**	1.622 (1.372–1.918)	**<0.0001**
MoCA score	2.666 (1.875–3.865)	**<0.0001**	2.652 (1.511–4.655)	**0.0007**
Imaging findings				
SLI	3.250 (2.108–5.042)	**<0.0001**		
CMBs	2.903 (1.803–4.710)	**<0.0001**		
DWMH	2.484 (1.680–3.734)	**<0.0001**	2.085 (1.111–3.911)	**0.022**
PVH	3.880 (2.438–6.498)	**<0.0001**	5.142 (2.430–10.880)	**<0.0001**
CS-EPVS	1.531 (1.163–2.033)	**0.0027**	1.558 (1.013–2.394)	**0.043**
BG-EPVS	2.969 (2.049–4.301)	**<0.0001**		
Total CSVD score	6.751 (4.585–10.30)	**<0.0001**	9.782 (3.336–28.683)	**<0.0001**

SCD, Subjective Cognitive Decline; OR, odds ratio; CI, confidence interval; y, years; m, months; Ref, reference; MDS-UPDRS, Movement Disorder Society Unified Parkinson’s Disease Rating Scale; MoCA, Montreal Cognitive Assessment; SLI, silent lacunar infarction; CMBs, cerebral microbleeds; DWMH, deep white matter hyperintensities; PVH, periventricular hyperintensities; CS-EPVS, enlarged perivascular spaces of centrum semioval; BG-EPVS, enlarged perivascular spaces of basal ganglia; CSVD, cerebral small vessel disease. Bold *p*-values indicate statistical significance at the 0.05 level.

In the multivariate analysis, we incorporated all CSVD markers along with clinical variables that showed significant associations with SCD. These variables included age, age at onset, males, education, disease duration, levodopa-equivalent dosage, presence of hypertension, MDS-UPDRS III score and MoCA score. The total burden of CSVD (OR = 9.782; 95% CI, 3.336–28.683) was identified as a strong independent predictor of SCD. Specific imaging markers, such as DWMH (OR = 2.085; 95% CI, 1.111–3.911), PVH (OR = 5.142; 95% CI, 2.430–10.880) and CS-EPVS (OR = 1.558; 95% CI, 1.013–2.394), remained significant predictors after adjusting for other variables (see Table 2).

**Figure 2:**
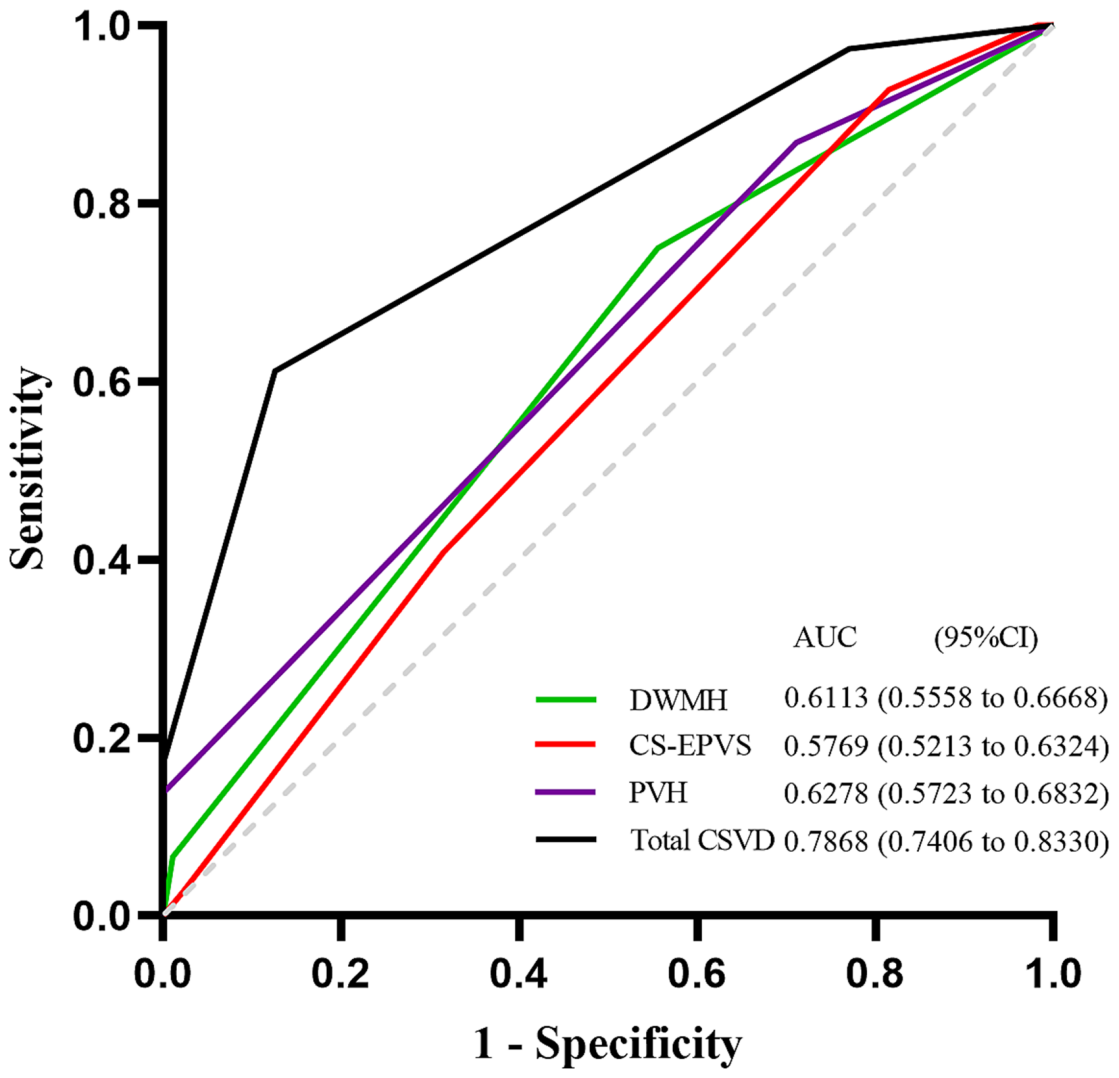
ROC curves for prediction of SCD in PD patients. ROC curves of the total burden of CSVD, DWMH, PVH and CS-EPVS in predicting SCD in PD patients. The AUC with 95% CI is provided. ROC, Receiver Operating Characteristic; SCD, Subjective Cognitive Decline; PD, Parkinson’s disease; CSVD, Cerebral Small Vessel Disease; DWMH, deep white matter hyperintensities; PVH, Periventricular Hyperintensities; CS-EPVS, enlarged perivascular spaces in centrum semiovale; AUC, Area Under the Curve; CI, Confidence Interval.

The diagnostic accuracy of the significant CSVD markers identified in the multivariate logistic regression analysis was assessed using the area under the curve (AUC) from the receiver operating characteristic (ROC) curves. Figure 2 illustrates the ROC curves for these markers. The total CSVD burden demonstrated the highest AUC value of 0.7868 (95% CI, 0.7406–0.8330), suggesting superior predictive accuracy for SCD in PD patients. The AUC values for DWMH, PVH, and CS-EPVS were 0.6113 (95% CI, 0.5558–0.6668), 0.6278 (95% CI, 0.5723–0.6832), and 0.5769 (95% CI, 0.5213–0.6324), respectively. The total CSVD burden showed a sensitivity of 0.612, specificity of 0.874, and accuracy of 0.78 when using a cut-off value of 1.5, resulting in a Youden’s index of 0.486. Comparatively, DWMH, PVH, and CS-EPVS exhibited varying degrees of sensitivity and specificity, with cut-off values optimizing their predictive performance (Supplementary Table S3).

**Figure 3:**
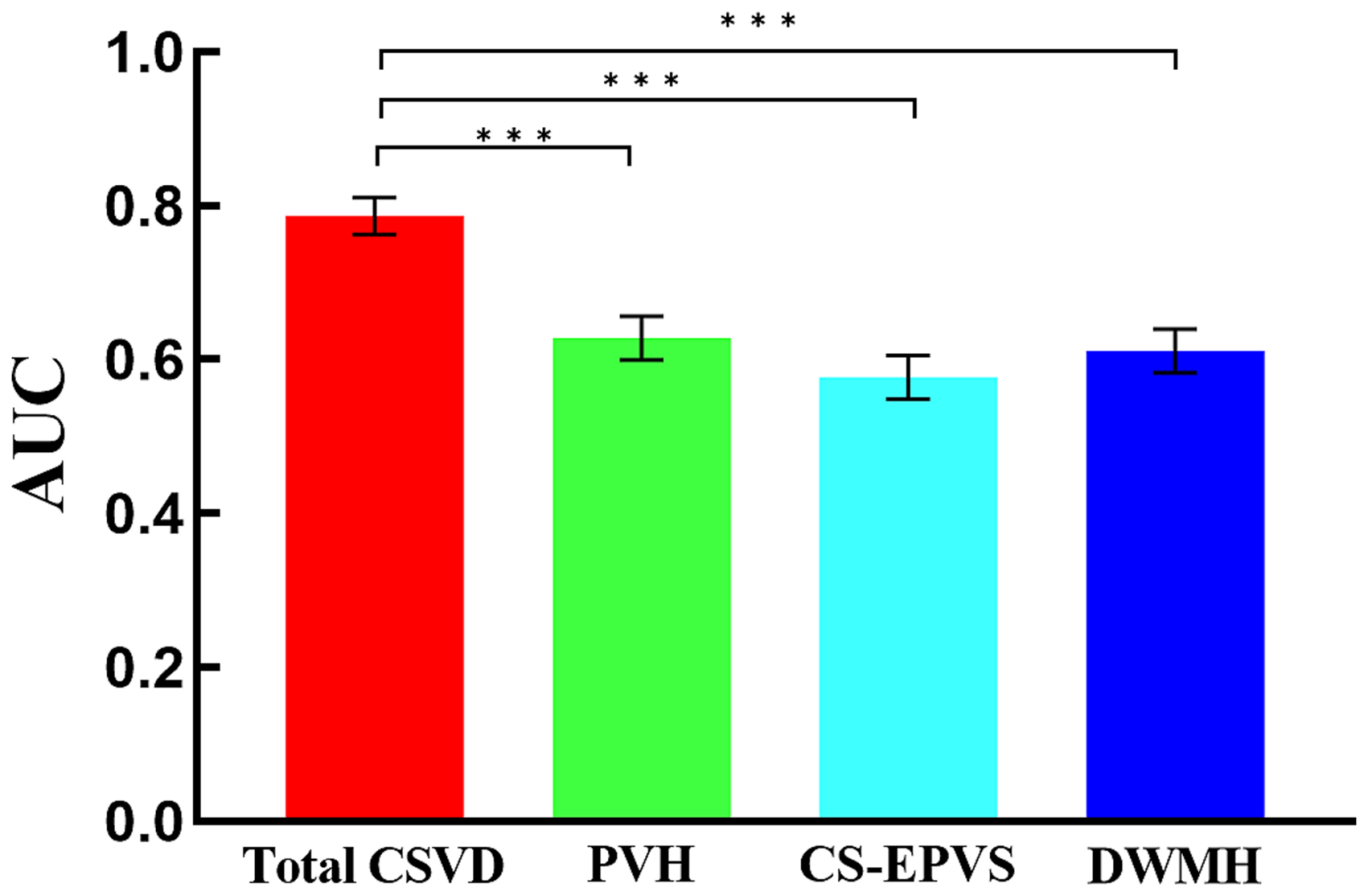
Comparative analysis of AUCs from ROC curves for different CSVD markers (***p < 0.001). Each bar represents the AUC of a corresponding ROC curve shown in Figure 2. AUC, Area Under the Curve; CI, Confidence Interval; ROC, Receiver Operating Characteristic; CSVD, Cerebral Small Vessel Disease.

The group comparison revealed that the total burden of CSVD demonstrated the highest AUC value (Figure 3, Supplementary Table S4), indicating its superior predictive accuracy for SCD.

## 4 Discussion

Our study offers valuable insights into the intricate relationship between CSVD and SCD in Parkinson’s disease, a connection that may signify an early stage in the spectrum of cognitive impairment in PD. The significant associations observed between CSVD markers and SCD underscore the importance of considering vascular factors in the early detection of cognitive changes in PD, aligning with the growing body of evidence that suggests a vascular contribution to cognitive impairment in neurodegenerative diseases (Toledo et al., 2013; Raz et al., 2016). These findings are in line with previous research that has highlighted the role of CSVD in cognitive impairment (Li et al., 2021; Chen et al., 2023; Wu et al., 2024). For instance, a previous study demonstrated that EPVS, as one of the characteristic manifestations of CSVD on MRI, may be associated with an increased risk of developing MCI and dementia in PD (Park et al., 2019).

The presence of SCD may precede the development of mild cognitive impairment (MCI) and dementia in PD, underscoring an urgent requirement for sensitive diagnostic tools capable of identifying the initial whispers of cognitive transformation (Huang et al., 2023; Flores-Torres et al., 2023; Hong et al., 2018). The collective evidence from our study intimates that the total burden of CSVD, as measured by MRI, could serve as a potential biomarker for the early identification of individuals at risk of cognitive decline in PD. This is in concordance with recent studies that have implicated CSVD in the pathogenesis of cognitive impairment in various neurological conditions, including PD (Fan et al., 2021; Chen et al., 2023; Cai et al., 2015; Arba et al., 2018).

The CSVD markers that remained significant predictors of SCD in our multivariate analysis, such as DWMH, PVH, and EPVS, are indicative of the microvascular changes that may contribute to cognitive impairment (Wardlaw et al., 2013; Benedictus et al., 2015; Blom et al., 2019). These imaging findings are consistent with the hypothesis that CSVD could disrupt the neural networks underlying cognitive function, leading to the subjective experience of cognitive decline. The disruption of these networks could result from ischemic changes, inflammation, and blood–brain barrier dysfunction, which are common pathological features of CSVD (Mustapha et al., 2019; Gao et al., 2022; Inoue et al., 2023).

While our study does not directly address the pathophysiological mechanisms linking CSVD and SCD, it is plausible that CSVD could lead to cognitive decline by causing ischemia, inflammation, and disruption of the blood–brain barrier. These pathologies may, in turn, affect the integrity of white matter tracts and neuronal function, leading to the clinical manifestations of SCD in PD patients. This is supported by studies that have shown a correlation between CSVD and cognitive impairment in PD, with CSVD markers such as lacunar infarcts and white matter hyperintensities being associated with an increased risk of cognitive decline (Suemoto et al., 2017; Jokinen et al., 2011; Sunwoo et al., 2014).

The area under the curve (AUC) from the receiver operating characteristic (ROC) curves demonstrated that the total burden of CSVD had the highest predictive accuracy for SCD in PD patients. This finding supports the utility of the total burden of CSVD as a diagnostic tool in clinical settings, potentially aiding in the early detection of cognitive impairment in PD. The predictive value of the total burden of CSVD is further emphasized by its ability to integrate multiple CSVD markers, providing a comprehensive assessment of CSVD burden.

However, our study has several limitations that should be acknowledged. The primary limitation of our study is its cross-sectional design, which limits our ability to establish causal relationships between CSVD and SCD in PD patients. Longitudinal studies are needed to confirm whether CSVD burden predicts the development of SCD and subsequent cognitive impairment in PD. Furthermore, the lack of a unified standard for defining SCD is a big problem. Although, the Cognitive Complaints Inventory (CCI) questionnaire and a threshold value of 3 were employed in our study, which is supported by previous researches (Thomas-Antérion et al., 2006; Yoo et al., 2020), there is a diversity of methods used in the literature to assess SCD (Weintraub et al., 2024; Siciliano et al., 2024). This variability in SCD assessment could impact the comparability of our findings with those from other studies and may influence the generalizability of our results. Therefore, a standardized approach to SCD evaluation is desired to facilitate more robust comparisons across studies and enhance the applicability of the findings in various contexts. Additionally, we recognize that affective symptoms, such as depression and anxiety, can significantly influence self-reported cognitive complaints. However, our study did not incorporate specific measures to account for these symptoms, which could have confounded the association between CSVD and SCD. In subsequent research, we intend to include assessments for depression and anxiety to control for their potential effects. Finally, our sample may not be fully representative of the broader PD population, suggesting a need for further research with diverse populations.

In conclusion, our study provides evidence that CSVD is closely associated with SCD in PD, and the total burden of CSVD may serve as a potential biomarker for early detection of cognitive decline in PD. These findings contribute to the growing understanding of the role of CSVD in cognitive impairment in PD and may have implications for the development of targeted interventions aimed at reducing CSVD burden and potentially slowing the progression of cognitive decline.

## Data Availability

The original contributions presented in the study are included in the article/Supplementary material, further inquiries can be directed to the corresponding author.
