# Supplementary material for: Total burden of cerebral small vessel disease predict subjective cognitive decline in patients with Parkinson’s disease
Source: Front Aging Neurosci. 2024 Nov 22;16:1476701. doi: 10.3389/fnagi.2024.1476701 (PMC11621090; doi:10.3389/fnagi.2024.1476701)
Supplement: Supplementary file 1 [file Table_1.DOCX]

The cognitive complaint interview

|  | Questions concerning the last 6 months | Response |
| --- | --- | --- |
| 1 | Have you observed a memory change during the last 6 months? | Yes/no |
| 2 | During the last 6 months, do you consider that your memory has been worse than the memory of your peers? | Yes/no |
| 3 | Do you record less recent events or have you heard your family say ‘I have already said so to you’? | Yes/no |
| 4 | Do you often forget appointments? | Yes/no |
| 5 | Do you often forget where things are left? | Yes/no |
| 6 | Do you have more difficulty finding your way in your neighborhood? Have you ever not recognized a route that your family thinks you have already gone? | Yes/no |
| 7 | Have you ever forgotten a whole event, even when the family gives you clues, details or pictures of the event? | Yes/no |
| 8 | Have you ever encountered difficulty finding particular words (except person names)? | Yes/no |
| 9 | Have you reduced your activities (social or leisure’s activities, association, papers and invoices) or asked your family to help you because you are afraid you may make a mistake? | Yes/no |
| 10 | Have you ever observed mood changes in term of apathy, blunted affect, inertia, loss of volition or interest for activities or persons? | Yes/no |

Ref: Catherine T. A. R., Sandrine H. M., Bernard L. (2006). The cognitive complaint interview (CCI). Psychogeriatrics 6 S18–S22
