# Supplementary material for: Total burden of cerebral small vessel disease predict subjective cognitive decline in patients with Parkinson’s disease
Source: Front Aging Neurosci. 2024 Nov 22;16:1476701. doi: 10.3389/fnagi.2024.1476701 (PMC11621090; doi:10.3389/fnagi.2024.1476701)
Supplement: Supplementary file 2 [file Table_2.DOCX]

| **Table S2 Analysis of the association between the clinical and neuroimaging characteristics and the SCD scores of patients with Parkinson’s disease** | | | | | | |
| --- | --- | --- | --- | --- | --- | --- |
|  | | Univariate analysis | |  | Multivariate analysis | |
|  | Coefficient | | *P* value |  | Coefficient | *P* value |
| **Demographic and clinical characteristics** | | | | | | |
| Age, y | 0.014 | | 0.1409 |  |  |  |
| Age at onset, y | **0.042** | | **<0.0001** |  | **0.027** | **0.0011** |
| Sex |  | |  |  |  |  |
| Female | Ref | |  |  | Ref |  |
| Male | **0.427** | | **0.0020** |  | **0.260** | **0.0216** |
| Education, y | **-0.131** | | **<0.0001** |  | **-0.074** | **<0.0001** |
| Disease duration, m | -0.008 | | 0.1465 |  |  |  |
| Levodopa-equivalent dose, mg | **0.002** | | **0.0016** |  | **0.001** | **0.0224** |
| Hypertension | **0.401** | | **0.0130** |  |  |  |
| Diabetes mellitus | 0.384 | | 0.3546 |  |  |  |
| Hyperlipidemia | -0.158 | | 0.1832 |  |  |  |
| Smoking | -0.130 | | 0.4697 |  |  |  |
| Drinking | 0.073 | | 0.7195 |  |  |  |
| MDS-UPDRS III score | **0.229** | | **<0.0001** |  | **0.144** | **<0.0001** |
| MoCA score | **0.627** | | **<0.0001** |  | **0.367** | **<0.0001** |
| **Imaging findings** | | | | | | |
| SLI | **0.703** | | **<0.0001** |  | **0.231** | **0.0165** |
| CMBs | **0.510** | | **0.0024** |  |  |  |
| DWMH | **0.467** | | **<0.0001** |  | **0.311** | **0.0021** |
| PVH | **0.676** | | **<0.0001** |  | **0.287** | **<0.0001** |
| CS-EPVS | **0.254** | | **0.0059** |  | **0.218** | **0.0172** |
| BG-EPVS | **0.555** | | **<0.0001** |  |  |  |
| Total CSVD score | **0.759** | | **<0.0001** |  | **0.485** | **0.0181** |
| **Abbreviations:** SCD, Subjective Cognitive Decline; OR, odds ratio; CI, confidence interval; y, years; m, months; Ref, reference; MDS-UPDRS, Movement Disorder Society Unified Parkinson’s Disease Rating Scale; MoCA, Montreal Cognitive Assessment; SLI, silent lacunar infarction; CMBs, cerebral microbleeds; DWMH, deep white matter hyperintensities; PVH, periventricular hyperintensities; CS-EPVS, enlarged perivascular spaces of centrum semioval; BG-EPVS, enlarged perivascular spaces of basal ganglia; CSVD, cerebral small vessel disease. | | | | | | |
