# Supplementary material for: Total burden of cerebral small vessel disease predict subjective cognitive decline in patients with Parkinson’s disease
Source: Front Aging Neurosci. 2024 Nov 22;16:1476701. doi: 10.3389/fnagi.2024.1476701 (PMC11621090; doi:10.3389/fnagi.2024.1476701)
Supplement: Supplementary file 3 [file Table_3.DOCX]

| **Table S3. Performance Metrics of CSVD Markers in Predicting SCD in PD Patients** | | | | | |
| --- | --- | --- | --- | --- | --- |
| CSVD Markers | Sensitivity | Specificity | Accuracy | Cut-off Value | Youden's Index |
| Total CSVD | 0.612 | 0.874 | 0.78 | 1.5 | 0.486 |
| DWMH | 0.75 | 0.444 | 0.55 | 0.5 | 0.194 |
| PVH | 0.868 | 0.289 | 0.498 | 0.5 | 0.157 |
| CS-EPVS | 0.928 | 0.185 | 0.453 | 1.5 | 0.113 |
| **Abbreviations:** CSVD, cerebral small vessel disease; PVH, periventricular hyperintensities; CS-EPVS, enlarged perivascular spaces of centrum semioval; DWMH, deep white matter hyperintensities. | | | | | |
