# Supplementary material for: Total burden of cerebral small vessel disease predict subjective cognitive decline in patients with Parkinson’s disease
Source: Front Aging Neurosci. 2024 Nov 22;16:1476701. doi: 10.3389/fnagi.2024.1476701 (PMC11621090; doi:10.3389/fnagi.2024.1476701)
Supplement: Supplementary file 4 [file Table_4.DOCX]

| **Table S4 AUC and standard error of the significant CSVD markers** | | |
| --- | --- | --- |
|  | AUC | standard error |
| Total CSVD | 0.7868 | 0.02357 |
| PVH | 0.6278 | 0.02828 |
| CS-EPVS | 0.5769 | 0.02832 |
| DWMH | 0.6113 | 0.02831 |
| **Abbreviations:** AUC, area under the curve; CSVD, cerebral small vessel disease; PVH, periventricular hyperintensities; CS-EPVS, enlarged perivascular spaces of centrum semioval; DWMH, deep white matter hyperintensities. | | |
